# Association Between Opioid Use Disorder and Healthcare Spending and Utilization in Emergency Surgical Patients: A Retrospective Analysis Using Commercial Claims

**DOI:** 10.1097/AS9.0000000000000568

**Published:** 2025-04-10

**Authors:** Anjali A. Dixit, Pooja A. Lagisetty, Michelle C. Odden, Mark C. Bicket, Keith Humphreys, Sean C. Mackey, Eric C. Sun

**Affiliations:** From the * Department of Anesthesiology, Perioperative and Pain Medicine, Stanford University School of Medicine, Stanford, CA; † Department of Medicine, University of Michigan School of Medicine, Ann Arbor, MI; ‡ Center for Clinical Management and Research, Ann Arbor Veterans Administration, Ann Arbor, MI; § Department of Epidemiology and Population Health, Stanford University School of Medicine, Stanford, CA; ‖ Overdose Prevention Engagement Network, Institute for Healthcare Innovation and Policy, University of Michigan School of Medicine, Ann Arbor, MI; ¶ Department of Anesthesiology, University of Michigan School of Medicine, Ann Arbor, MI; # Department of Psychiatry and Behavioral Sciences, Stanford University School of Medicine; Stanford, CA; ** Department of Neurology; Stanford University School of Medicine, Stanford, CA; †† Department of Health Policy, Stanford University School of Medicine, Stanford, CA.

## Abstract

**Objective:**

To estimate the association between opioid use disorder (OUD) and healthcare spending and utilization in emergency surgical patients, and to evaluate whether the use of opioid agonist treatment (OAT) modifies this relationship.

**Background:**

Surgical patients with OUD are susceptible to challenging postoperative pain management and relapse. Their healthcare spending and utilization estimates may justify perioperative system optimization efforts.

**Methods:**

We identified 142,726 patients who underwent 1 of 14 surgeries between January 1, 2016 and December 31, 2021. We then estimated the association between OUD and primary outcomes (spending during the surgical admission and in the 1–90 days postdischarge) and secondary outcomes (measures of healthcare utilization). We further evaluated whether the use of OAT modified the relationship between OUD and outcomes.

**Results:**

Those with *versus* without OUD had no difference in spending during the surgical admission [−1%; 95% confidence interval (CI) = −7% to +4%; *P* = 0.644]. However, in the postdischarge period, those with OUD had 38% higher spending (95% CI = 17% to 62%; *P* < 0.001), translating to $2,560 (95% CI = $786–$4,333; *P* = 0.005) in incremental spending. Hospital length-of-stay was not different in those with OUD incidence risk ratio (IRR) = 0.99; 95% CI = 0.92–1.05; *P* = 0.668), but all measures of postdischarge utilization were elevated (number of postdischarge inpatient days, IRR = 1.90; 95% CI = 1.39–2.58; *P* < 0.001; 30-day inpatient readmission, IRR = 1.30; 95% CI = 1.06–1.60; *P* = 0.013; and 30-day emergency department utilization (IRR = 1.28; 95% CI = 1.10–1.48; *P* = 0.001). Point estimates for all postdischarge outcomes were lower in those with OUD who used OAT versus those with OUD who did not use OAT.

**Conclusions:**

Emergency surgical patients with OUD had higher healthcare spending and utilization following discharge compared to those without OUD, implying an elevated risk of complications. Optimizing preoperative use of OAT may facilitate perioperative optimization and cost savings.

## INTRODUCTION

Opioid use disorder (OUD) affects approximately 7.6 million individuals in the United States.^1^ Patients with OUD and other substance use disorders, which are increasingly managed as chronic diseases, comprise a disproportionate share of surgical patients.^2,3^ Although chronic disease is common in surgical populations,^4^ and some chronic conditions have a robust evidence base for perioperative care,^5,6^ there is little evidence to guide the optimization of perioperative management of patients with OUD.

Surgical patients with OUD pose a clinical challenge because of the tension between managing their pain—often with opioid medication—and minimizing the risk of relapse.^7^ Those with OUD frequently have a higher tolerance for opioid medications, which can lead to challenges in achieving perioperative pain control. Further complicating perioperative care for such patients, OUD is increasingly managed with opioid agonist treatment (OAT) medication—specifically, methadone or buprenorphine—which can compromise the pain-relieving effect of other opioid medications.^8^ Given these complexities in clinical management, patients with OUD who undergo surgery may have difficult-to-control postoperative pain, one of the main predictors of 30-day emergency department (ED) utilization and inpatient readmissions after surgery.^9^ Some studies have found an increased risk of surgical and other postoperative complications for patients with OUD in the immediate postoperative period (ie, before discharge) but did not evaluate the use of OAT as a modifiable risk factor.^10–14^ They also did not address the potential for biased estimates due to patients with severe OUD being less likely to be offered elective surgery.^15^

Economic evaluations have found immense societal costs associated with a diagnosis of OUD^16^ and large lifetime savings from treatment with OAT.^17^ However, these estimates have not specifically evaluated surgical patients. Given projected growth in the number of patients with OUD and the proportion who will increasingly use OAT,^18^ there remains a need to quantify healthcare spending and utilization in surgical patients with OUD and evaluate whether OAT use influences their postoperative (including postdischarge) course. Such information can help justify the cost of systems-level changes aimed at optimizing the perioperative management of surgical patients with OUD.

We used a nationwide sample of commercially insured patients who underwent emergency inpatient surgeries to estimate the association between a preoperative diagnosis of OUD and perioperative healthcare spending and utilization from the date of admission to 90 days after discharge. We focused on emergency surgeries to minimize potential selection bias in the assignment of surgery for those with severe OUD. We also assessed whether using OAT in the presurgical period modified the association between OUD and perioperative healthcare spending. We hypothesized that (1) surgical patients with OUD would have increased healthcare spending and utilization in the perioperative period compared to surgical patients without OUD, and (2) use of OAT would attenuate the relationship between OUD and these outcomes.

## METHODS

### Data, Setting, and Participants

We used healthcare claims from the Merative MarketScan Commercial Database^19^ of commercial claims for patients enrolled in large employer-sponsored plans nationwide—for patients aged ≥13 and <65 years. We included adolescents given that they are at high risk for OUD and its sequelae.^14^ Our sample included those who underwent any of 14 selected inpatient, emergency surgeries between January 1, 2016 and December 31, 2021. Additional detail, including detail on the identification of emergency surgery, is provided in Supplemental Digital Content 1 and 2, http://links.lww.com/AOSO/A489.

### Opioid Use Disorder

Patients were categorized as having OUD if they had at least 2 International Classification of Diseases 10th edition (ICD-10) diagnosis codes for opioid use, dependence, or abuse during the 12 months before surgical admission.^20,21^ Patients in the comparison group had zero relevant diagnosis codes in the previous year.

### Healthcare Spending

The primary outcomes were per-member composite healthcare spending for insurer-paid and patient-paid medical care (inpatient, ED, and outpatient) during the following 2 time periods: (1) from date of admission through date of discharge, capturing the cost of surgery as well as contiguous preoperative and postoperative inpatient care, and (2) in the 1 to 90 days following date of discharge, capturing the cost of postdischarge medical care. To attenuate the influence of outliers, we winsorized these values at the 1st and 99th percentile, assigning patients who fell outside those bounds to the value found at the 1st and 99th percentile, respectively. All dollar values were converted to 2021 dollars using the Consumer Price Index.^22^

### Healthcare Utilization

We evaluated the following secondary utilization outcomes: (1) hospital length-of-stay in days, (2) number of days with inpatient claims in the 1 to 90 days after discharge, (3) any inpatient readmission within 30 days of discharge, and (4) any utilization of ED care within 30 days of discharge.

### Preoperative Utilization of Opioid Agonist Therapy

We assessed the preoperative utilization of OAT as an effect modifier in the relationship between surgery and our primary outcomes. We identified OAT using prescription drug fills for buprenorphine, or cCurrent procedural terminology / healthcare common procedure coding system procedural codes associated with methadone or buprenorphine administration. Using a claims-based algorithm previously defined by others,^23^ we assigned patients to OAT if they had at least 50% of days covered in the 30 days before the date of surgery. We excluded buprenorphine specifically formulated for chronic pain.

### Additional Variables

To adjust for potential confounding, we included a robust set of covariates selected a priori. We included age and sex as defined using enrollment files. Given higher baseline healthcare utilization^24^ in patients with OUD, we included a continuous variable representing total medical (ie, inpatient, outpatient, and ED) spending the 180 days before admission. Given the increased risk of comorbid pain^25^ requiring treatment with opioids in patients with OUD, and known elevated costs and healthcare utilization in patients with chronic pain,^26^ we also included continuous variables representing total nonbuprenorphine and nonmethadone oral morphine milligram equivalents filled in the 1 to 30, 31 to 90, and 91 to 365 days before admission. We included indicator variables for Elixhauser^27^ comorbidities and additional psychiatric diagnoses^28^ including other nonopioid and nontobacco substance use disorders (Supplemental Digital Content 3, http://links.lww.com/AOSO/A489), given the high rate of comorbid psychiatric illness^29^ in patients with OUD. Finally, we included year- and surgery-level fixed effects to account for secular trends and for differences in spending within surgery types, respectively.

### Statistical Analysis

We calculated descriptive statistics and absolute standardized differences (SDs) across covariates for those who had a diagnosis of OUD and those who did not, with an SD greater than 0.1 representing a meaningful difference between groups.

To estimate our primary outcome of spending during the surgical admission, we fit a multivariable generalized linear model with the gamma family and log link, choosing this approach because the dependent variable was positive and right-skewed.^30^ This approach provided the ratio in spending for those with OUD relative to those without OUD (ie, a spending ratio), which is easily understood across different types of surgeries that are typically reimbursed at different average amounts. The spending ratio can be interpreted as the multiplicative value in patients with OUD compared to those without OUD. For ease of interpretation, we converted these to the percent difference in spending relative to the non-OUD comparison group. The primary independent variable was an indicator representing whether the patient had OUD, and we further adjusted for the other covariates and fixed effects described above. To evaluate whether the utilization of OAT modified the relationship between OUD and spending, we estimated a second model with an additional coefficient for the product of OUD and OAT.

In estimating our primary outcome of spending during days 1 to 90 postdischarge, we accounted for the small proportion of patients (approximately 8%) who had no costs during this period, to avoid underestimating the association between OUD and the outcome. We used a two-step approach, as has been done in other healthcare studies that include a large proportion of observations with a value of zero (Supplemental Digital Content 4, http://links.lww.com/AOSO/A489).^31^ As a post hoc analysis, we also estimated the absolute dollar amount in 90-day postdischarge spending associated with a diagnosis of OUD (ie, the adjusted marginal—or incremental—effect of OUD on postdischarge spending), accounting for both steps of the model as described above.^32^ To estimate our secondary outcomes, we used negative binomial regression given overdispersion, with the same covariates and fixed effects as above.

We calculated robust standard errors for all models and considered a two-sided *P* < 0.05 to be statistically significant. Per expert guidance,^33^ analyses of secondary outcomes were not adjusted for multiple comparisons.

### Sensitivity Analyses

We conducted several prespecified sensitivity analyses of the primary outcomes. First, we expanded the study population by including patients who disenrolled from a MarketScan plan before the 90-day endpoint in the main analysis and adjusted for their early disenrollment using inverse probability of attrition weights.^34^ We included this analysis given that patients with OUD are at higher risk for death and unemployment^35^ (and therefore, losing employer-sponsored health insurance) relative to the general population. Second, we redefined OUD less specifically, requiring only one relevant ICD-10 diagnosis codes in the past 365 days, rather than 2.

### Subgroup Analysis

We conducted 3 prespecified subgroup analyses. We repeated our primary analyses for patients with OUD stratified by whether they: (1) did *versus* did not undergo an emergency surgery with expected postoperative pain (eg, those who underwent transurethral interventions and endoscopies would not generally be expected to have postoperative pain), (2) did *versus* did not have other co-occurring nontobacco substance use disorders, and (3) did *versus* did not have evidence of long-term prescription opioid use in the 90 to365 days before admission.

This study was approved by the Stanford University Institutional Review Board with a waiver of consent and followed Strengthening the Reporting of Observational Studies in Epidemiology reporting guidelines. All analyses were conducted using STATA version 17.0.

## RESULTS

Characteristics of the population are presented in Table 1 and in Supplemental Digital Content 5, http://links.lww.com/AOSO/A489, and stratified by patients with and without a preoperative diagnosis of OUD. Our final sample included 142,726 surgical patients, 686 (0.5%) of whom met criteria for OUD. Of these, 117 (17.1%) used OAT in the 30 days before hospital admission. Those with OUD were more likely to have comorbid disease, including mental health diagnoses, other nonopioid and nontobacco substance use disorders, and higher healthcare spending during the 180 days before surgical admission. They were also more likely to have preoperative prescription opioid use, corresponding with reports that patients with OUD are often prescribed opioids for management of comorbid disease, due to inadequate monitoring by prescribers or due to lack of resources to taper prescription opioid use in the context of OUD.^36,37^

**TABLE 1.** Selected Characteristics of the Study Population, by OUD Diagnosis

	Diagnosis of Opioid Use Disorder		
	No	Yes	
N	142,040	686	Standardized Mean Difference
Demographic characteristics (%)			
Sex, female	61142 (43.0)	299 (43.6)	0.01
Sex, male	80898 (57.0)	387 (56.4)	0.01
Age, years			0.24
13–18 years	2936 (2.1)	<16	
19–36 years	21196 (14.9)	<120	
37–45 years	20182 (14.2)	114 (16.6)	
46–55 years	40249 (28.3)	210 (30.6)	
56–65 years	57477 (40.5)	244 (35.6)	
Preoperative healthcare spending			
Total inpatient and outpatient healthcare spending in 180 days before admission (mean [SD])	10,981 (23,209)	27,591 (40,605)	0.50
Preoperative opioid utilization			
Total oral morphine equivalents filled in 1–30 days before admission (mean [SD])	162 (5171)	4309 (66,358)	0.09
Total oral morphine equivalents filled in 31–90 days before admission (mean [SD])	267 (6,553)	8886 (132,727)	0.09
Total oral morphine equivalents filled in 91–365 days before admission (mean [SD])	355 (8720)	10,951 (133,887)	0.11
Selected comorbidities (%)			
Alcohol abuse	3573 (2.5)	102 (14.9)	0.45
Depression	20,940 (14.7)	322 (46.9)	0.74
Psychoses	383 (0.3)	19 (2.8)	0.21
Anxiety disorder	22,040 (15.5)	313 (45.6)	0.69
Attention deficient hyperactivity disorder	2880 (2.0)	31 (4.5)	0.14
Post-traumatic stress disorder	1576 (1.1)	45 (6.6)	0.29
Bipolar disorder	1942 (1.4)	45 (6.6)	0.27
Nonopioid, nontobacco, and nonalcohol substance use disorder	899 (0.6)	139 (20.3)	0.68
Selected surgeries			
Laparoscopic appendectomy	14,046 (9.9)	38 (5.5)	0.16
Laparoscopic cholecystectomy	25,252 (17.8)	116 (16.9)	0.02
Operative management of traumatic hip fracture	39,348 (27.7)	224 (32.7)	0.11
Colectomy for diverticulitis	21,654 (15.2)	75 (10.9)	0.13
Transurethral intervention for urolithiasis/nephrolithiasis	21,654 (15.2)	75 (10.9)	0.13

Table 1 presents baseline demographic characteristics, preoperative spending and opioid utilization, Elixhauser comorbidities and additional psychiatric disorders, and surgical types, comparing patients with a diagnosis of opioid use disorder (OUD) to those without using standardized mean differences (SMD), with SMD>0.1 reflective of meaningful differences between groups. For continuous variables, means are presented with standard deviations in parentheses. For binary variables, the percent of patients out of the total is presented in parentheses. Some cell sizes have been suppressed for patient privacy in accordance with our data use agreement with Merative MarketScan. For full list of comorbidities and included surgeries, see Supplemental Digital Content http://links.lww.com/AOSO/A489.

Median spending for surgical admissions was $29,491 (IQR $20,194–$42,276). 91.5% of patients had nonzero spending in days 1 to 90 postdischarge; of those, median postdischarge spending was $1,978 (IQR $519–$6,369). Median length of stay for the hospital admission was 2 days (IQR 1–4). In the postdischarge period, of the 9.4% of patients with any inpatient utilization, the median number of inpatient days was 4 (IQR 2–7). Incidence of 30-day inpatient readmissions was 5.9%, and the incidence of 30-day ED utilization was 10.9%.

**FIGURE 1.**
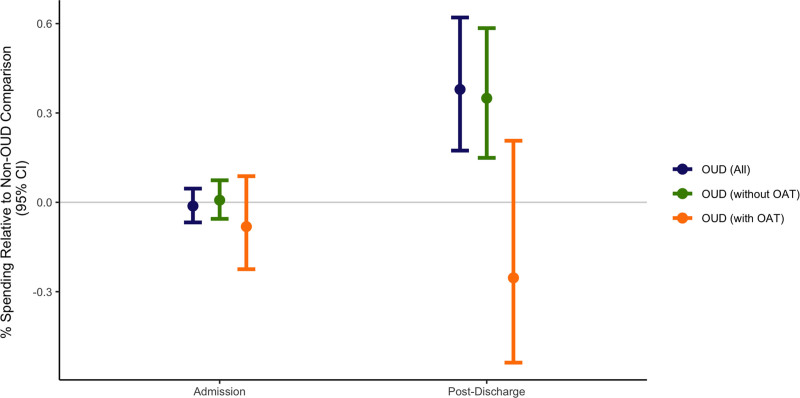
Admission and 90-day postdischarge spending in patients with OUD. Figure 1 presents results of multivariable models estimating spending during surgical admission and in the 90-day postdischarge period for patients with OUD compared to those without OUD. Coefficients were first estimated using generalized linear models with the gamma family and log link for all patients with OUD relative to the comparison group, adjusted for demographic characteristics, preoperative healthcare spending and opioid utilization, comorbidities, and year and surgery fixed effects. Coefficients were then re-estimated with the addition of a term representing the product of OUD and the use of OAT, which provided individual estimates for patients with OUD who did and did not use OAT. All coefficients for these models were spending ratios, which have been converted here to percentages (ie, percent higher or lower spending relative to the non-OUD comparison group) for ease of interpretation. Postdischarge spending was specified as a two-part model, given that roughly 8% of patients had zero spending in the postdischarge period. Odds of any spending in the postdischarge period was 2.47 for those with OUD relative to those without (95% CI = 1.28–4.77; *P* = 0.007). The estimates reported in the figure represent estimates from the second part of the two-part model, which was restricted to the proportion of patients with non-zero spending. Corresponding coefficients, 95% confidence intervals, and *P* values are presented in Table 2. ED, emergency department; IP, inpatient; OAT, opioid agonist treatment; OUD, opioid use disorder.

In analyses adjusted for demographic characteristics, preoperative healthcare spending, and comorbidities (Figure 1 and Table 2), there was no significant difference in spending during the surgical admission for those with OUD (–1%, 95% CI = –7% to 4%; *P* = 0.644). In the 90-day postdischarge period, those with OUD had 2.47 times the odds of having nonzero spending compared to those without OUD (95% CI = 1.28–4.77, *P* = 0.007); of patients with nonzero spending, those with OUD had 38% higher spending than the reference group (95% CI = 17%–62%; *P* < 0.001). Post hoc analysis estimated the incremental effect of OUD on postdischarge spending to be $2,560 (95% CI = $786–$4,333; *P* = 0.005).

**TABLE 2.** Estimates with Main Model Specifications

	Model 1:	Model 2:	
	OUD (All) Estimate (95% CI) *P*	OUD (Without OAT) Estimate (95% CI) *P*	OUD (With OAT) Estimate (95% CI) P*
Primary outcomes			
Admission spending	–1% (–7% to 5%) *P* = 0.672	1% (–6% to 7%) *P* = 0.827	–8% –22% to 9% *P* = 0.324
Postdischarge spending†	38% (17%–62%) *P* <0.001	35% (15%–58%) *P* <0.001	–25% (–54%–21%) *P* = 0.233
Secondary outcomes			
Hospital length-of-stay	IRR = .99 (0.92–1.05) *P* = 0.668	IRR = 1.06 (0.99–1.13) *P* = 0.094	IRR = 0.94 (0.78–1.13) *P* = 0.510
Postdischarge inpatient days	IRR = 1.90 (1.39–2.58) *P* < 0.001	IRR = 1.88 (1.38–2.55) *P* < 0.001	IRR = 0.41 (0.44–4.48) *P* = 0.561
30-day inpatient readmission	IRR = 1.30 (1.06–1.60) *P* = 0.013	IRR = 1.36 (1.10–1.68) *P* = 0.005	IRR = 0.63 (0.25–1.60) *P* = 0.333
30-day ED utilization	IRR = 1.28 (1.10–1.48) *P* = 0.001	IRR = 1.31 (1.12–1.53) *P* = 0.001	IRR = 0.55 (0.32–0.96) *P* = 0.034*

Table 2 presents results of multivariable models estimating relative risks or incidence risk ratios of spending and utilization for patients with and without OUD. Model 1 adjusted for demographic characteristics, preoperative healthcare spending and opioid utilization, comorbidities, and year and surgery fixed effects. Model 2 included all the aforementioned variables and further included an interaction term for OUD and OAT, which provided stratified estimates for patients with OUD who did or did not use OAT. Coefficients for the primary (spending) outcomes were spending ratios, which have been converted here to percentages (ie, percent higher or lower spending relative to the non-OUD comparison group) for ease of interpretation. Coefficients for the secondary outcomes are incidence rate ratios, which can be interpreted on the multiplicative scale.

* *P* value in this column represents a formal test for interaction, that is, whether OAT modifies the relationship between OUD and the outcome.

† Postdischarge spending was specified as a two-part model, given that roughly 8% of patients had zero spending in the postdischarge period. Odds of any spending in the postdischarge period was 2.47 for those with OUD relative to those without (95% CI = 1.28–4.77; *P* = 0.007). The numbers reported in the table represent estimates from the second part of the two-part model, which was restricted to the proportion of patients with nonzero spending.

**FIGURE 2.**
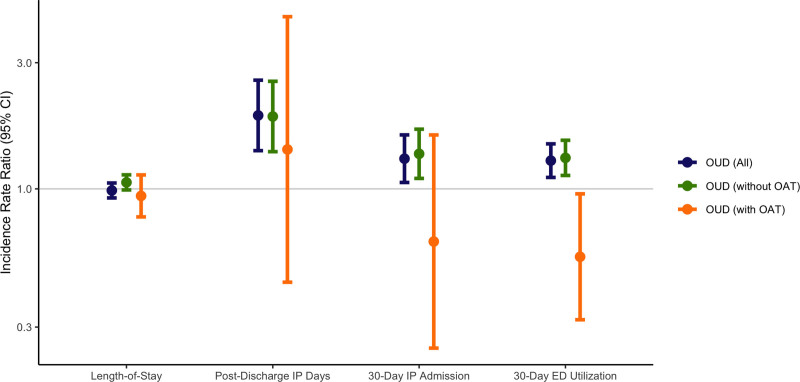
Healthcare utilization in patients with OUD. Figure 2 presents the results of multivariable negative binomial models estimating incidence rate ratios of measures of healthcare utilization for patients with OUD compared to those without OUD. Coefficients were first estimated using negative binomial models for all patients with OUD relative to the comparison group, adjusted for demographic characteristics, preoperative healthcare spending and opioid utilization, comorbidities, and year and surgery fixed effects. Coefficients were then re-estimated with the addition of a term representing the product of OUD and the use of OAT, which provided individual estimates for patients with OUD who did and did not use OAT. Corresponding coefficients, 95% confidence intervals, and *P* values are presented in Table 2. ED, emergency department; IP, inpatient; OAT, opioid agonist treatment; OUD, opioid use disorder.

Adjusted analyses of secondary outcomes (Figure 2 and Table 2) found that hospital length of stay during the surgical admission was not significantly different in those with OUD compared to those without [incidence risk ratio (IRR) = 0.99 times the length of stay (95% CI = 0.92–1.05; *P* = 0.668)]. However, measures of postdischarge utilization were all significantly different: those with OUD had 1.90 times the number of postdischarge inpatient days (95% CI = 1.39–2.58; *P* < 0.001), 1.30 times the risk of 30-day inpatient readmission (95% CI = 1.06–1.60; *P* = 0.013), and 1.28 times the risk of 30-day ED utilization (95% CI = 1.10–1.48; *P* = 0.001).

Formal tests of interaction found that OAT modified the relationship between OUD and 30-day ED utilization, with those using OAT having an attenuated risk (OUD without OAT: IRR = 1.31; 95% CI = 1.12–1.53; OUD with OAT: IRR = 0.55; 95% CI = 0.32–0.96; *P* value for interaction = 0.034). Other formal tests of interaction did not find that preoperative utilization of OAT modified the relationship between OUD and any of the primary or other secondary outcomes. However, point estimates for all outcomes were lower for those with OUD who used OAT relative to those who did not, with notably lower estimates for postdischarge spending, postdischarge inpatient days, and 30-day inpatient readmissions (Figures 1 and 2), although these estimates were imprecise. Findings were robust to alternate specifications of the analytic cohort and the outcome (Supplemental Digital Content 6, http://links.lww.com/AOSO/A489).

Subgroup analyses found that expected postoperative pain modified the relationship between OUD and postdischarge spending, with postoperative pain magnifying spending (OUD without expected postoperative pain: –4%; 95% CI = –22% to 18%; OUD with expected postoperative pain: 58%; 95% CI = 20%–108%, *P* value for interaction = 0.001). Co-occurring nontobacco substance use disorders and long-term prescription opioid use were not significant modifiers in the relationship between OUD and the primary outcomes (Table 3).

**TABLE 3.** Subgroup Analyses for Primary Outcomes

	Admission Spending Estimate (%) (95% CI) *P*	Post-Discharge Spending Estimate (%) (95% CI) *P*
Subgroup 1:		
OUD without expected postoperative pain	–9 (–24 to 11) *P* = 0.354	–4 (–22 to 18) *P* = 0.684
OUD with expected postoperative pain	9 (–11 to 33) *P* = 0.398	58 (20–108) *P* = 0.001*
Subgroup 2:		
OUD without other substance use disorder	1 (–5 to 7) *P* = 0.735	45 (19–77) *P*<0.001
OUD with other substance use disorder	–11 (–23 to 3) *P* = 0.120	–6 (–35 to 36) *P* = 0.741*
Subgroup 3:		
OUD without long-term prescription opioid use	–4 (–12 to 5) *P* = 0.351	57 (17–111) *P* = 0.003
OUD with long-term prescription opioid use	7 (–5 to 19) *P* = 0.262	–19 (–43 to 15) *P* = 0.245*

Table 3 presents results from exploratory subgroup analyses. We repeated our primary analyses first for patients stratified by whether they did or did not undergo an emergency surgery with expected postoperative pain (ie, those who underwent transurethral interventions and endoscopies would not generally be expected to have postoperative pain). We then examined the primary outcomes in patients with OUD who did or did not have other co-occurring nontobacco substance use disorders. Finally, we examined the primary outcomes in patients who did or did not have evidence of long-term prescription opioid use (ie, in the 90–365 days before admission). All estimates are relative to a reference group of patients without OUD. *P value in this column represents a formal test for interaction, ie, whether OAT modifies the relationship between OUD and the outcome.

## DISCUSSION

Spending during the initial surgical admission did not differ between emergency surgical patients with or without OUD but was approximately 38% higher in those with OUD in the 90-day postdischarge period, corresponding to approximately $2560 in average additional spending per patient across all surgery types. During this postdischarge period, patients with OUD also had 1.2 to 1.9 times higher rates of healthcare utilization across multiple measures: total number of inpatient days, 30-day readmissions, and 30-day ED utilization. Because our models included surgery-specific fixed effects, the resulting estimates reflect differences in patients with versus without OUD within each particular surgery type and therefore account for surgery-specific complication rates that would be expected following a given type of surgery.

These findings uphold our hypothesis that surgical patients with OUD have increased healthcare spending compared to those without OUD. While others have found increased healthcare utilization in surgical subgroups with OUD, their analyses were limited to the immediate postoperative period before discharge and focused on specific surgical subpopulations and/or those undergoing mostly elective surgery, which could be biased given providers’ reluctance to offer surgery to patients with severe OUD.^10–14^ We circumvented these limitations by focusing on patients undergoing emergency surgical procedures and evaluating both admission and postdischarge outcomes. In addition, our findings advance the literature by identifying significantly different measures of post-discharge utilization for those with OUD, implying differences in postdischarge recovery trajectories. In line with this, our exploratory subgroup analysis found that undergoing a procedure with expected postoperative pain was a modifier in the relationship between OUD and postdischarge spending, with more painful surgeries associated with higher spending.

While it may be reasonable for patients with OUD to require more frequent outpatient care after undergoing surgery (eg, for pain monitoring or OAT adjustments), the elevated risks of 30-day inpatient admission and ED utilization imply that patients with OUD may also have a higher risk of unexpected postsurgical outcomes. 30-day readmissions in surgical patients are commonly due to pain, postoperative infections, sepsis, and exacerbation of underlying chronic conditions such as diabetes, and a large proportion of readmissions are potentially preventable^9,38^; however, specific reasons for readmission in those with OUD have not yet been evaluated. OUD-specific complications in the postdischarge period may include uncontrollable pain due to inadequate pain management (eg, due to stigma^39^ or given a high baseline tolerance to opioids), development of prolonged postoperative opioid use and/or misuse, and relapse or overdose.^40^

Findings related to our second hypothesis—that utilization of OAT would modify the relationship between OUD and outcomes—were mixed. 30-day ED utilization was significantly lower in patients with OUD who used OAT versus those who did not. Formal tests for interaction were not significant for other spending or utilization outcomes; however, our estimates were limited by small sample sizes. Lower point estimates for those using OAT for all postdischarge spending and utilization outcomes imply that there may in fact be a decrease in spending and utilization for patients with OUD who use OAT versus those who do not. To the extent that there is a difference in these outcomes, our findings corroborate the work of others who have found that adherence to buprenorphine as OAT in the nonsurgical setting is associated with lower healthcare utilization and lower risk of inpatient admissions.^41^ Patients using OAT may be more likely to have a higher-quality transition of care after their surgical admission, particularly given that they are likely to have relationships with behavioral health, pain medicine, and/or addiction medicine specialists.^42,43^ These healthcare practitioners may help patients with OUD cope with the stress of undergoing emergency surgery, understand the importance and limitations of OAT in the setting of acute pain, and advocate for their patients to receive closer postsurgical follow-up or additional pain medication. Our findings are preliminary and warrant additional research with larger sample sizes, particularly focusing on transitions of care and pain trajectories following surgery.

### Limitations

Our study has important limitations. First, our analysis was limited to a commercially insured population. The commercially insured population represents approximately 40% of nonelderly individuals in the United States with OUD. Our findings may not apply to patients enrolled in Medicaid, who make up another 40% of those with OUD and often have more risk factors for complications such as mental health comorbidities and significant social disadvantages, including homelessness.^44^ Second, because our analysis focused on emergency surgical patients to avoid potential selection bias in the assignment of surgery to patients with severe OUD, specific spending and healthcare utilization estimates from this study may not apply to elective surgical populations. However, the findings suggest that patients with OUD scheduled for elective surgery may benefit from OUD-specific support through the perioperative period. Third, claims-based definitions of OUD can underestimate its true prevalence (ie, misclassify patients into the non-OUD comparison group)—which could have led to underestimated differences between groups—and can include some patients with chronic pain who have a physical dependence on prescribed opioids but may not meet Diagnostic and Statistical Manual 5th edition criteria for moderate to severe OUD. Fourth, the majority of patients using OAT in our population used buprenorphine rather than methadone, which is typical in commercially insured populations but may not reflect other populations. Buprenorphine may be offered along with other healthcare services meant to support individuals with OUD; thus, our OAT-specific estimates may represent the combined effects of these interventions. Claims-based diagnoses of OUD and utilization of OAT may reflect patients’ inherent engagement with the medical system, health literacy, and level of social support or access to high-quality care; thus, findings from this study may reflect differences in healthcare utilization and spending due to both clinical and other patient-level characteristics.

This study suggests that patients with OUD are at high risk for complications after surgery, which results in additional healthcare expenditures, and that the use of OAT may decrease the risk of these outcomes. A patient-centered, harm-reduction approach aimed at supporting patients with OUD through the perioperative setting, including optimizing OAT use in the perioperative period, may promote patient health and lead to cost savings for healthcare systems.

## Supplementary Material
