# Supplementary material for: Association Between Opioid Use Disorder and Healthcare Spending and Utilization in Emergency Surgical Patients: A Retrospective Analysis Using Commercial Claims
Source: Ann Surg Open. 2025 Apr 10;6(2):e568. doi: 10.1097/AS9.0000000000000568 (PMC12185090; doi:10.1097/AS9.0000000000000568)
Supplement: Supplementary file 1 [file as9-6-e568-s001.pdf]

**Supplemental Digital Content**

**Supplemental Digital Content 1: Additional Detail and Flowchart Depicting Study Sample Creation**

Cohort creation for this study is depicted below. We limited the initial sample to patients who underwent any of the 14 selected emergency surgery types and who: (1) were enrolled in non-capitated, non-health management organization (HMO) plans, given that this study investigates payments, which could be obscured in capitated and/or HMO plans, (2) had prescription drug benefits and mental health and substance abuse coverage provided by their insurance plan. Emergency surgeries were defined using Current Procedural Terminology (CPT®) codes associated with inpatient hospitalizations and an emergency room visit (based on an emergency room-specific revenue code) 0-5 days before surgery (see **Supplemental Digital Content 2**).

Next, we restricted the cohort to those who: (1) did not have another hospitalization in the 30 days prior to the date of surgery, given that OAT utilization was defined in the 30 days before admission for this study and could not be accurately measured during inpatient stays with our data, (2) were continuously enrolled for 365 days before admission and 90 days after discharge, allowing for identification of preoperative comorbidities and postoperative spending and utilization patterns, (3) did not have metastatic cancer, (4) did not exceed the 99th percentile in hospital length-of-stay, given that these patients were likely to be extreme outliers in postoperative surgical or medical complications, and (5) did not have negative total spending values in the preoperative, admission, and/or postoperative periods as these likely represented errors in the claims. To optimize the specificity of the group of patients with OUD, and further the group of those who had OUD but did not use OAT, we excluded (1) 4 patients who used OAT for less than 50% proportion of days covered, and (2) 187 patients with only 1 diagnosis code for OUD in the previous 365 days.

Of note, there were no patients in our sample who used long-acting injectable naltrexone in the 30 days prior to date of surgery.

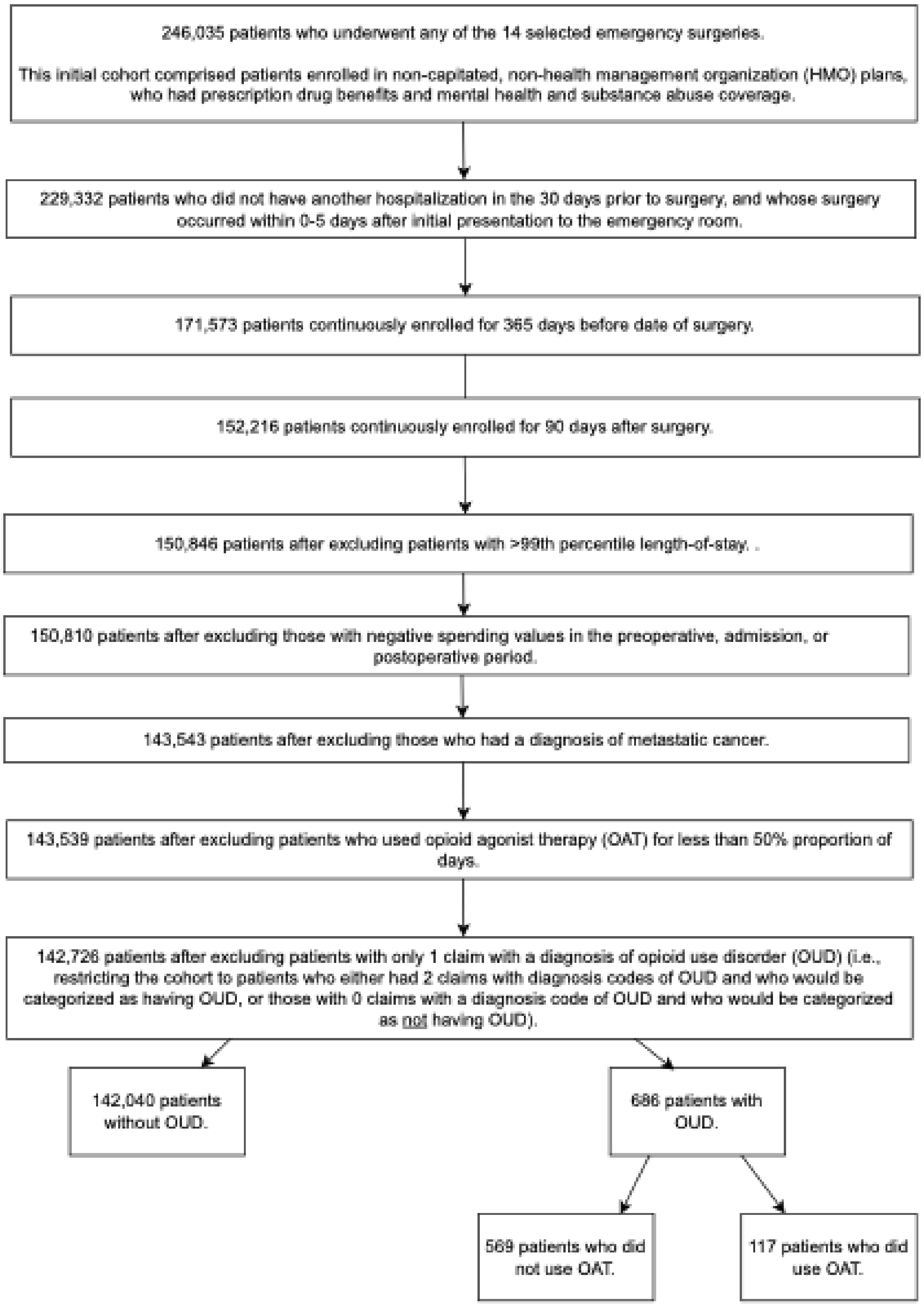
246,035 patients who underwent any of the 14 selected emergency surgeries.
This initial cohort comprised patients enrolled in non-capitated, non-health management organization (HMO) plans, who had prescription drug benefits and mental health and substance abuse coverage.
229,332 patients who did not have another hospitalization in the 30 days prior to surgery, and whose surgery occurred within 0-5 days after initial presentation to the emergency room.
171,573 patients continuously enrolled for 365 days before date of surgery.
152,216 patients continuously enrolled for 90 days after surgery.
150,846 patients after excluding patients with >99th percentile length-of-stay. .
150,810 patients after excluding those with negative spending values in the preoperative, admission, or postoperative period.
143,543 patients after excluding those who had a diagnosis of metastatic cancer.
143,539 patients after excluding patients who used opioid agonist therapy (OAT) for less than 50% proportion of days.
142,726 patients after excluding patients with only 1 claim with a diagnosis of opioid use disorder (OUD) (i.e., restricting the cohort to patients who either had 2 claims with diagnosis codes of OUD and who would be categorized as having OUD, or those with 0 claims with a diagnosis code of OUD and who would be categorized as not having OUD).
142,040 patients without OUD.
686 patients with OUD.
569 patients who did not use OAT.
117 patients who did use OAT.

**Supplemental Digital Content 2: Current Procedural Terminology Codes Used to Define Emergency Surgeries**

| **Type of Surgery** | **CPTs**[a] |
|---|---|
| Laparoscopic appendectomy[1,2] | 44950<br>44970<br>44960 |
| Laparoscopic cholecystectomy[3] | 47562<br>47563<br>47564<br>47600<br>47605<br>47610<br>47612<br>47620<br>47630 |
| Operative management of traumatic hip fracture[4] | 27125<br>27130<br>27230<br>27232<br>27235<br>27236<br>27246<br>27248<br>73530 |

| | |
|---|---|
| Colectomy for diverticulitis[5] | 44110<br>44111<br>44130<br>44139<br>44140<br>44141<br>44143<br>44144<br>44145<br>44156<br>44147<br>44150<br>44151<br>44155<br>44156<br>44157<br>44158<br>44160<br>44320<br>44187<br>44188<br>44204<br>44205<br>44206<br>44207<br>44208<br>44210<br>44211<br>44212<br>44213<br>44227<br>44238 |
| Operative management of adhesive small bowel obstruction[6] | 44180<br>44005 |
| Operative management of ovarian torsion | 58661<br>58662<br>58670 |
| Operative management of testicular torsion | 54640<br>54650<br>54692 |
| Operative management of ectopic pregnancy[7] | 59120<br>59121<br>59130<br>59135<br>59136<br>59140<br>59150<br>59151<br>58770<br>58673<br>58700<br>58720<br>49320<br>58661<br>58679 |

| | |
|---|---|
| Operative management of incarcerated or strangulated hernia[8] | 49521<br>49553<br>49557<br>49561<br>49566<br>49572<br>49587<br>49650<br>49653<br>49655<br>49657 |
| Laparoscopic or open repair of perforated peptic or duodenal ulcer[9] | 43631<br>43632<br>43633<br>43625<br>43659<br>44238<br>49329<br>43840<br>44602<br>44603<br>49000<br>49905 |
| Transurethral intervention for nephrolithiasis | 52005<br>52310<br>52332<br>52352<br>52353<br>52356<br>50590<br>52317<br>52318 |
| Upper endoscopy for foreign body removal | 43215<br>43247 |
| Upper endoscopy for management of bleeding peptic or duodenal ulcer[10] | 43227<br>43255<br>44366<br>44378 |

| | |
|---|---|
| Aneurysm Repair[11] | 0001T<br>0002T<br>0033T<br>0035T<br>0036T<br>0038T<br>0039T<br>0040T<br>0078T<br>0079T<br>0080T<br>0081T<br>33877<br>33880<br>33881<br>34800<br>34802<br>34803<br>34804<br>34805<br>34830<br>34831<br>34832<br>75952<br>75953 |

Emergency surgeries were defined as having any of the above procedural codes, plus a claim for emergency department care (based on revenue codes) in the 0-5 days before date-of-surgery.
[a]The X symbol following a decimal in the ICD-10 codes represents a wildcard (i.e., either no subsequent character, or any alphanumeric character).

**Supplemental Digital Content 3: Additional Information about Comorbidities Included as Covariates**

Psychiatric comorbidities included alcohol use disorder, depression, psychoses, anxiety, post-traumatic stress disorder, attention deficit hyperactivity disorder, bipolar disorder, and other non-tobacco substance use disorders (i.e., non-alcohol, non-opioid, and non-tobacco substance use disorders). ICD-10 codes for psychiatric disorders aside from alcohol use disorder, depression and psychoses were based on code lists previously supplied by others.[12] We excluded the Elixhauser comorbidity for drug use as this would be collinear with the primary exposure of interest. Patients were categorized as having the comorbidity if they had a relevant diagnosis code in the 365 days before admission.

**Supplemental Digital Content 4: Additional Information on Two-Step Model for Estimating Post-Discharge Spending**

To estimate our primary outcome of spending during the 1-90 days post-discharge, we used a two-step approach to account for the small proportion of patients (approximately 8%) who had no costs during the post-discharge period, which could lead to underestimating the true association between OUD and the outcome. The two-step approach has been done in other health care policy studies that include a large proportion of observations with a value of zero.[13] The first part involved estimating a multivariable logistic regression model in which the dependent binary variable represented whether a patient did or did not have non-zero costs in the post-discharge period. The second part replicated the multivariable generalized linear model as described above but restricted to the patients with non-zero spending in the post-discharge period. Both models adjusted for all covariates and fixed effects described in the main manuscript.

**Supplemental Digital Content 5: Characteristics of the Study Population, by OUD DIagnosis**

| | Diagnosis of Opioid Use Disorder | | |
|---|---|---|---|
| | No | Yes | Standardized mean difference |
| **N** | 142,040 | 686 | |
| **Demographic Characteristics (%)** | | | |
| Sex, female | 61142 (43.0) | 299 (43.6) | 0.01 |
| Sex, male | 80898 (57.0) | 387 (56.4) | 0.01 |
| Age, years | | | 0.24 |
| 13-18 years | 2936 (2.1) | <16 | |
| 19 to 36 years | 21196 (14.9) | <120 | |
| 37 to 45 years | 20182 (14.2) | 114 (16.6) | |
| 46 to 55 years | 40249 (28.3) | 210 (30.6) | |
| 56 to 65 years | 57477 (40.5) | 244 (35.6) | |
| **Preoperative Healthcare Spending** | | | |
| Total Inpatient and Outpatient Healthcare Spending in 180 Days before Admission (mean (SD)) | 10,981 (23,209) | 27,591 (40,605) | 0.50 |
| **Preoperative Opioid Utilization** | | | |
| Total Oral Morphine Equivalents Filled in 1-30 Days before Admission (mean (SD)) | 162 (5,171) | 4,309 (66,358) | 0.09 |
| Total Oral Morphine Equivalents Filled in 31-90 Days before Admission (mean (SD)) | 267 (6,553) | 8,886 (132,727) | 0.09 |
| Total Oral Morphine Equivalents Filled in 91-365 Days before Admission (mean (SD)) | 355 (8,720) | 10,951 (133,887) | 0.11 |
| **Comorbidities (%)** | | | |
| AIDS / HIV | 446 (0.3) | <16 | 0.02 |
| Alcohol abuse | 3573 (2.5) | 102 (14.9) | 0.45 |
| Blood loss anemia | 3403 (2.4) | 34 (5.0) | 0.14 |
| Other anemia | 8850 (6.2) | 89 (13.0) | 0.23 |
| Cardiac arrhythmias | 18716 (13.2) | 157 (22.9) | 0.26 |
| Congestive heart failure | 3821 (2.7) | 34 (5.0) | 0.12 |
| Coagulopathy | 5502 (3.9) | 58 (8.5) | 0.19 |
| Chronic pulmonary disease | 17269 (12.2) | 148 (21.6) | 0.25 |
| Depression | 20940 (14.7) | 322 (46.9) | 0.74 |
| Fluid and electrolyte disorders | 22657 (16.0) | 223 (32.5) | 0.39 |
| Hypertension, complicated | 5917 (4.2) | 48 (7.0) | 0.12 |
| Hypertension, uncomplicated | 61727 (43.5) | 345 (50.3) | 0.14 |
| Hyperthyroidism | 17002 (12.0) | 113 (16.5) | 0.13 |
| Liver disease | 17440 (12.3) | 138 (20.1) | 0.21 |
| Lymphoma | 750 (0.5) | <16 | 0.01 |
| Obesity | 43239 (30.4) | 203 (29.6) | 0.02 |
| Other neurologic disorders | 3782 (2.7) | 77 (11.2) | 0.34 |

| | | | |
|---|---|---|---|
| Paralysis | 700 (0.5) | <16 | 0.11 |
| Psychoses | 383 (0.3) | 19 (2.8) | 0.21 |
| Chronic peptic ulcer disease | 3083 (2.2) | 40 (5.8) | 0.19 |
| Pulmonary circulation disorder | 1744 (1.2) | 19 (2.8) | 0.11 |
| Renal failure | 5167 (3.6) | 47 (6.9) | 0.15 |
| Rheumatoid arthritis | 6038 (4.3) | 92 (13.4) | 0.33 |
| Solid tumor | 12338 (8.7) | 42 (6.1) | 0.10 |
| Valvular disease | 5679 (4.0) | 38 (5.5) | 0.07 |
| Weight loss | 4715 (3.3) | 72 (10.5) | 0.29 |
| Anxiety disorder | 22040 (15.5) | 313 (45.6) | 0.69 |
| Attention deficient hyperactivity disorder | 2880 (2.0) | 31 (4.5) | 0.14 |
| Post-traumatic stress disorder | 1576 (1.1) | 45 (6.6) | 0.29 |
| Bipolar disorder | 1942 (1.4) | 45 (6.6) | 0.27 |
| Non-opioid, non-tobacco, and non-alcohol substance use disorder | 899 (0.6) | 139 (20.3) | 0.68 |
| **Type of Surgery** | | | |
| Laparoscopic appendectomy | 14046 (9.9) | 38 (5.5) | 0.16 |
| Laparoscopic cholecystectomy | 25252 (17.8) | 116 (16.9) | 0.02 |
| Operative management of traumatic hip fracture | 39348 (27.7) | 224 (32.7) | 0.11 |
| Colectomy for diverticulitis | 21654 (15.2) | 75 (10.9) | 0.13 |
| Operative management of adhesive small bowel obstruction | 3681 (2.6) | 25 (3.6) | 0.06 |
| Operative management of ovarian torsion | 1671 (1.2) | <16 | 0.06 |
| Operative management of testicular torsion[c] | 41 (0.0) | <16 | 0.02 |
| Operative management of ectopic pregnancy | 7224 (5.1) | 21 (3.1) | 0.10 |
| Operative management of incarcerated or strangulated hernia | 4098 (2.9) | <16 | 0.04 |
| Laparoscopic or open repair of perforated peptic or duodenal ulcer[c] | 6868 (4.8) | 53 (7.7) | 0.12 |
| Upper endoscopy for management of bleeding peptic or duodenal ulcer | 3014 (2.1) | 35 (5.1) | 0.16 |
| Upper endoscopy for foreign body removal | 421 (0.3) | <16 | 0.09 |
| Transurethral intervention for urolithiasis or nephrolithiasis | 21654 (15.2) | 75 (10.9) | 0.13 |
| Operative management of aortic aneurysm or dissection | 736 (0.5) | <16 | 0.01 |

**Supplemental Digital Content 5** presents baseline demographic characteristics, preoperative spending and opioid utilization, Elixhauser comorbidities and additional psychiatric disorders, and surgical types, comparing patients with a diagnosis of opioid use disorder (OUD) to those without using standardized mean differences (SMD), with SMD>0.1 reflective of meaningful differences between groups. For continuous variables, means are presented with standard deviations in parentheses. For binary variables, the percent of patients out of the total is presented in parentheses.
Some cell sizes have been suppressed for patient privacy in accordance with our data use agreement with Merative™ MarketScan®.

**Supplemental Digital Content 6: Sensitivity Analyses for Primary Outcomes**

| | **Sensitivity Analysis 1: Inverse Probability of Attrition Weighting** Estimate (%) (95% CI) p-value | **Sensitivity Analysis 2: Respecified Definition of OUD** Estimate (%) (95% CI) p-value |
|---|---|---|
| **Admission Spending** | | |
| OUD (All) | -2 (-7 to +4) p=0.498 | 0 (-4 to +4) p=0.918 |
| OUD (no OAT) | +1 (-6 to +7) p=0.844 | +1 (-3 to +5) p=0.783 |
| OUD (with OAT) | -8 (-23 to +9) p=0.326** | -3 (-16 to +13) p=0.726** |
| **Post-Discharge Spending*** | | |
| OUD (All) | +37 (+17 to +61) p<0.001 | +33 (+20 to +49) p<0.001 |
| OUD (no OAT) | +35 (+15 to +58) p<0.001 | +28 (+15 to +42) p<0.001 |
| OUD (with OAT) | -25 (-54 to +20) p=0.228** | -13 (-42 to +32) p=0.519** |

**Supplemental Digital Content 6** presents sensitivity analyses for the two primary outcomes. The first sensitivity analysis expanded the study population to include patients who disenrolled from a MarketScan plan before the 90-day endpoint in the main analysis. We adjusted for their early disenrollment using inverse probability of attrition weights.[14]

The second sensitivity analysis repeated the main analysis but respecified the exposure variable (OUD) using a less specific definition requiring only one – rather than two – relevant ICD-10 diagnosis code in the 365 days prior to surgical admission.

*Analysis restricted to 92% of patients with non-zero post-discharge spending. Adjusted odds of non-zero post-discharge spending were 2.43 for those with versus without OUD (95% CI 1.26 to 4.70, p=0.008) for Sensitivity Analysis 1, and 2.10 (95% CI 1.37 to 3.24, p=0.001) for Sensitivity Analysis 2.

**P-value represents a formal test for interaction, i.e., whether OAT modifies the relationship between OUD and the outcome

**References for Supplemental Digital Content**


1. Harbaugh CM, Lee JS, Hu HM, et al. Persistent Opioid Use Among Pediatric Patients After Surgery. *Pediatrics*. Jan 2018;141(1)doi:10.1542/peds.2017-2439
2. Loehrer AP, Leech MM, Weiss JE, et al. Association of Cost Sharing With Delayed and Complicated Presentation of Acute Appendicitis or Diverticulitis. *JAMA Health Forum*. Sep 2021;2(9):e212324. doi:10.1001/jamahealthforum.2021.2324
3. Lois A, Fennern E, Cook S, Flum D, Davidson G. Patterns of care after cholecystostomy tube placement. *Surg Endosc*. May 2022;36(5):2778-2785. doi:10.1007/s00464-021-08562-3
4. Montgomery JR, Neiman PU, Brown CS, et al. Sources of Postacute Care Episode Payment Variation After Traumatic Hip Fracture Repair Among Medicare Beneficiaries: Cross-Sectional Retrospective Study. *Ann Surg Open*. Dec 2022;3(4):e218. doi:10.1097/AS9.0000000000000218
5. Simianu VV, Fichera A, Bastawrous AL, et al. Number of Diverticulitis Episodes Before Resection and Factors Associated With Earlier Interventions. *JAMA Surg*. Jul 1 2016;151(7):604-10. doi:10.1001/jamasurg.2015.5478
6. Carmichael SP, 2nd, Kline DM, Mowery NT, Miller PR, 3rd, Meredith JW, Hanchate AD. Geographic Variation in Operative Management of Adhesive Small Bowel Obstruction. *J Surg Res*. Jun 2023;286:57-64. doi:10.1016/j.jss.2022.12.040
7. Wall-Wieler E, Shover CL, Hah JM, Carmichael SL, Butwick AJ. Opioid Prescription and Persistent Opioid Use After Ectopic Pregnancy. *Obstet Gynecol*. Sep 2020;136(3):548-555. doi:10.1097/AOG.0000000000004015
8. Senkowski C, Savarise M, Roth JS, Nagle J. Hernia repair and complex abdominal wall reconstruction. Accessed Nov 15, 2023. https://www.facs.org/for-medical-professionals/news-publications/news-and-articles/bulletin/2017/04/hernia-repair-complex-abdominal-wall-reconstruction/
9. Jayaraman SS, Allen R, Feather C, Turcotte J, Klune JR. Outcomes of Laparoscopic vs Open Repair of Perforated Peptic Ulcers: An ACS-NSQIP Study. *J Surg Res*. Sep 2021;265:13-20. doi:10.1016/j.jss.2021.02.030
10. Quan S, Frolkis A, Milne K, et al. Upper-gastrointestinal bleeding secondary to peptic ulcer disease: incidence and outcomes. *World J Gastroenterol*. Dec 14 2014;20(46):17568-77. doi:10.3748/wjg.v20.i46.17568
11. Newton ER, Akerman AW, Strassle PD, Kibbe MR. Association of Fluoroquinolone Use With Short-term Risk of Development of Aortic Aneurysm. *JAMA Surg*. Mar 1 2021;156(3):264-272. doi:10.1001/jamasurg.2020.6165
12. Larochelle MR, Lodi S, Yan S, Clothier BA, Goldsmith ES, Bohnert ASB. Comparative Effectiveness of Opioid Tapering or Abrupt Discontinuation vs No Dosage Change for Opioid Overdose or Suicide for Patients Receiving Stable Long-term Opioid Therapy. *JAMA Netw Open*. Aug 1 2022;5(8):e2226523. doi:10.1001/jamanetworkopen.2022.26523
13. Deb P, Norton EC. Modeling Health Care Expenditures and Use. *Annu Rev Public Health*. Apr 1 2018;39:489-505. doi:10.1146/annurev-publhealth-040617-013517
14. Weuve J, Tchetgen Tchetgen EJ, Glymour MM, et al. Accounting for bias due to selective attrition: the example of smoking and cognitive decline. *Epidemiology*. Jan 2012;23(1):119-28. doi:10.1097/EDE.0b013e318230e861
